# Crystal and mol­ecular structures of two silver(I) amidinates, including an unexpected co-crystal with a lithium amidinate

**DOI:** 10.1107/S2056989016017680

**Published:** 2016-11-15

**Authors:** Sida Wang, Nicole Harmgarth, Phil Liebing, Frank T. Edelmann

**Affiliations:** a Chemisches Institut der Otto-von-Guericke-Universität Magdeburg, Universitätsplatz 2, 39106 Magdeburg, Germany

**Keywords:** crystal structure, lithium, silver, amidinate, alkynylamidinate, co-crystal

## Abstract

The silver(I) amidinates bis­[μ-*N* ^1^,*N* ^2^-bis­(propan-2-yl)benzamidinato-κ^2^ *N* ^1^:*N* ^2^]disilver(I), [Ag_2_(C_13_H_19_N_2_)_2_] or [Ag{PhC(N^*i*^Pr)_2_}]_2_ (**1**), and bis­(μ-*N* ^1^,*N* ^2^-di­cyclohexyl-3-cyclo­propyl­propynamidinato-κ^2^ *N* ^1^:*N* ^2^)disilver(I), [Ag_2_(C_18_H_27_N_2_)_2_] or [Ag{*cyclo*-C_3_H_5_–C≡C–C(NCy)_2_}]_2_ (**2a**), exist as centrosymmetric dimers with a planar Ag_2_N_4_C_2_ ring and a common linear coordination of the metal atoms in the crystalline state. Moiety **2a** forms a co-crystal with the related lithium amidinate, namely bis­(μ-*N* ^1^,*N* ^2^-di­cyclo­hexyl-3-cyclo­propyl­propynamidinato-κ^2^ *N* ^1^:*N* ^2^)disilver(I) bis­(μ-*N* ^1^,*N* ^2^-di­cyclo­hexyl-3-cyclo­propyl­propynamidinato-κ^3^ *N* ^1^,*N* ^2^:*N* ^1^)bis­(tetra­hydro­furan-κ*O*)lithium(I) toluene monosolvate, [Ag_2_(C_18_H_27_N_2_)_2_][Li_2_(C_18_H_27_N_2_)_2_(C_4_H_8_O)_2_]·C_7_H_8_ or [Ag{*cyclo*-C_3_H_5_–C≡*C*–*C*(NCy)_2_}]_2_[Li{*cyclo*-C_3_H_5_–C≡C–C(NCy)_2_}(THF)]_2_·C_7_H_8_, composed as **2a** × **2b** × toluene. The lithium moiety **2b** features a typical ladder-type dimeric structure with a distorted tetra­hedral coordination of the metal atoms. In the silver(I) derivatives **1** and **2a**, the amidinate ligand adopts a μ-κ*N*:κ*N*′ coordination, while it is a μ-κ*N*:κ*N*:κ*N*′-coordination in the case of lithium derivative **2b**.

## Abstract 2

In the dimeric silver(I) amidinates [Ag{*R*C(N*R*′)_2_}]_2_ (*R* = Ph, cyclo­propyl­alkynyl; *R*′ = Cy, ^*i*^Pr), a centrosymmetric planar Ag_2_N_4_C_2_ ring with strictly linearly coordinated silver ions is present. [Ag{*cyclo*-C_3_H_5_—C≡C–C(NCy)_2_}]_2_ forms a 1:1 co-crystal with the related lithium derivative [Li{*cyclo*-C_3_H_5_—C≡C–C(NCy)_2_}(THF)]_2_, in which the lithium component exhibits a typical dimeric ladder structure.

## Chemical context   

Anionic *N*-chelating donor ligands such as amidinates, [*R*C(N*R*)_2_]^−^, and guanidinates, [*R*_2_NC(N*R*)_2_]^−^, have gained tremendous importance in various fields of organometallic and coordination chemistry over the past two decades. Formally, amidinate anions are the nitro­gen analogues of the carboxyl­ate anions, while guanidinates are similarly related to the carbamates. However, in contrast to the carboxyl­ates and carbamates, the steric properties of amidinates and guanidinates can be widely tuned through the use of different substituents, both at the outer nitro­gen atoms as well as at the central carbon atom of the NCN unit. Both types of *N*-chelating ligands are often regarded as ‘steric cyclo­penta­dienyl equivalents’ (Bailey & Pace, 2001 ▸; Collins, 2011 ▸; Edelmann, 2008 ▸, 2013 ▸). Meanwhile, amidinato and guanidinato complexes are known for virtually every metallic element in the Periodic Table ranging from lithium to uranium (Edelmann, 2008 ▸, 2009 ▸, 2012 ▸, 2013 ▸; Trifonov, 2010 ▸). Alkyl-substituted amidinate and guanidinate complexes of various metals have also been established as ALD and MOCVD precursors for the deposition of thin layers of metals, metal oxides, metal nitrides *etc.* (Devi, 2013 ▸). The most important starting mat­erials in this field are lithium amidinates and guanidinates. Lithium amidinates are normally prepared in a straightforward manner by addition of lithium alkyls to *N,N′*-di­organo­carbodi­imides in a 1:1 molar ratio, while lithium guanidinates are formed when lithium-*N,N*-di­alkyl­amides are added to *N,N′*-diorganocarbodi­imides (Stalke *et al.*, 1992 ▸; Aharonovich *et al.*, 2008 ▸; Chlupatý *et al.*, 2011 ▸; Nevoralová *et al.*, 2013 ▸; Hong *et al.*, 2013 ▸). On the other hand, silver(I) amidinates and guanidinates (Archibald *et al.*, 2000 ▸; Lim *et al.*, 2003 ▸; Whitehorne *et al.*, 2011 ▸; Lane *et al.*, 2014 ▸) are of significant importance as potential precursors for vapor deposition processes (Lim *et al.*, 2003 ▸; Whitehorne *et al.*, 2011 ▸), as precursors for silver nanoparticles (Cure *et al.*, 2015 ▸), or as inter­mediates in silver-catalyzed amidination and guanylation reactions (Pereshivko *et al.*, 2011 ▸; Okano *et al.*, 2012 ▸; Li *et al.*, 2015 ▸).
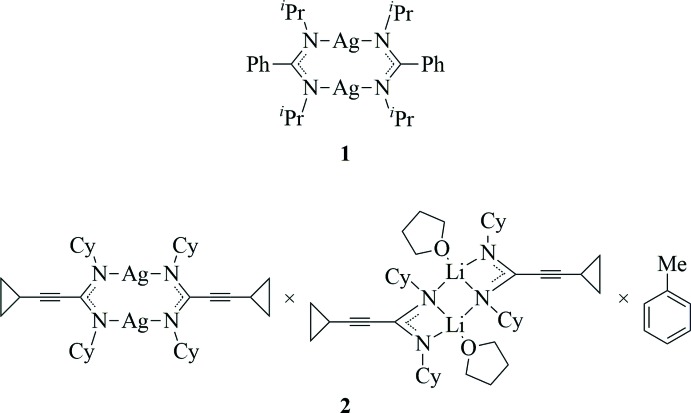


We report here the structural characterization of two silver(I) amidinates, namely [Ag{PhC(N^*i*^Pr)_2_}]_2_ (**1**), and the unexpected co-crystal (**2)**, composed as [Ag{*cyclo*-C_3_H_5_—C≡C—C(NCy)_2_}]_2_ (**2a**) × [Li{*cyclo*-C_3_H_5_—C≡C—C(NCy)_2_}(THF)]_2_ (**2b**) × toluene (Cy = cyclo­hex­yl).

## Structural commentary   

**Figure 1:**
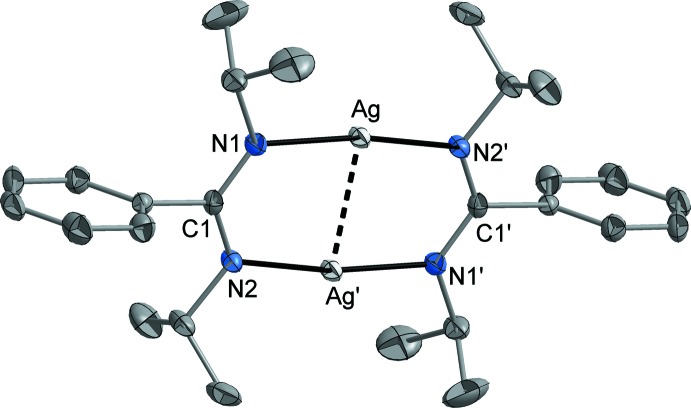
The mol­ecular structure of compound **1**. Displacement ellipsoids are drawn at the 50% probability level and H atoms have been omitted for clarity. [Symmetry code: (′) 2 − *x*, 2 − *y*, 1 − *z*.]

**Figure 2:**
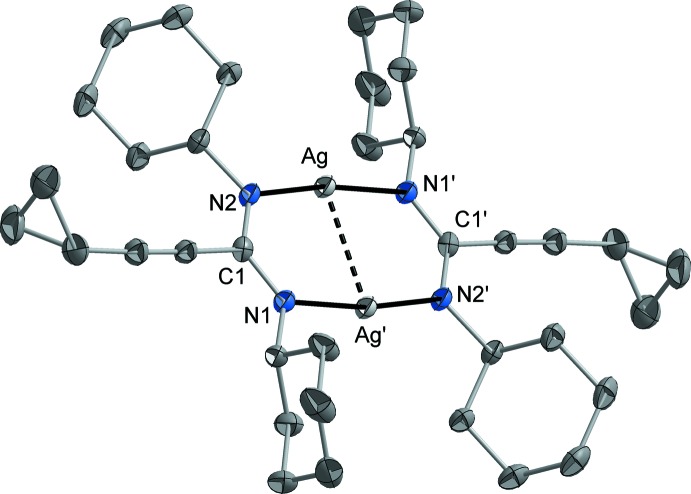
The mol­ecular structure of moiety **2a**, determined from the co-crystal **2**. Displacement ellipsoids are drawn at the 50% probability level and H atoms have been omitted for clarity. [Symmetry code: (′) 2 − *x*, 2 − *y*, 1 − *z*.]

Silver(I) compound **1** (Fig. 1 ▸) and silver moiety **2a** (Fig. 2 ▸): Both silver(I) complexes exist as centrosymmetric dimers in the crystalline state. Compound **1** crystallizes without any solvent, and the mol­ecular structure of moiety **2a** was determined from the co-crystal **2** (**2a** × **2b** × toluene). In both **1** and **2a**, each of the two N atoms of the amidinate ligand coordinates to one Ag atom (coordination mode μ-κ*N*:κ*N′*), and the Ag atoms adopt an almost linear coordination [**1**: N—Ag—N 170.58 (7)°; **2a**: N—Ag—N 170.66 (5)°] by two N atoms of two symmetry-related amidinate ligands, leading to centrosymmetric dimers in each case. The Ag—N separations are very similar in both structures [**1**: 2.0959 (16) and 2.0965 (16) Å, **2a**: 2.0908 (15) and 2.0916 (14) Å]. An *sp* ^2^ hybridization can be assigned to the N atoms since the coordination environment is almost trigonal–planar. The C—N separations within the amidinate NCN fragment are virtually equal [**1**: twice 1.322 (3) Å, **2a**: 1.329 (2) and 1.331 (2) Å], indicating a typical delocalization of the negative charge. Through the mentioned connectivity pattern, a strictly planar C_2_N_4_Ag_2_ eight-membered ring with a short Ag⋯Ag contact is built [**1**: 2.6604 (3) Å, **2a**: 2.6838 (3) Å]. This constitution might be supported by some attractive *d* ^10^–*d* ^10^ inter­action between the Ag atoms that have been frequently discussed in the literature (for a review, *e.g.* see: Jansen, 1987 ▸). The mol­ecular structures of the here discussed compounds are closely related to those of the most previously described copper(I) and silver(I) amidinates, namely [Cu_2_{*R*C(N*R*′)_2_}_2_] (*R*, *R*′ = Me, ^*n*^Bu; Li *et al.*, 2005 ▸) and [*M*_2_{MeC(N^*i*^Pr)_2_}_2_] (*M* = Cu, Ag). However, in the case of Ag{MeC(N^*i*^Pr)_2_}, also a trimeric structure [Ag_3_{MeC(N^*i*^Pr)_2_}_3_] was observed (Lim *et al.*, 2003 ▸). The bond lengths and angles involving the Ag atoms, *viz*. Ag—N and Ag—Ag distances and N—Ag—N angles, in the compounds discussed herein resemble those observed in the previously reported dimeric silver(I) amidinates. A dimerization or oligomerization under formation of linear N—*M*—N units is also typical for a broad ensemble of copper(I) and silver(I) complexes with other anionic nitro­gen ligands, *e.g.* [Cu_4_(N*R*_2_)_4_] (*e.g. R* = Me, Gambarotta *et al.*, 1987 ▸; *R* = Et, Hope & Power, 1984 ▸; *R* = SiMe_3_, James *et al.*, 1998 ▸), [Ag_4_(N(SiMe_3_)_2_}_4_] and [Ag_3_(*N*,*N*,*N′*,*N′*-tetra­methyl­piperid­yl)_3_] (Hitchcock *et al.*, 1996 ▸), [Cu_2_Tl_2_(ThioSila)_2_] and [Ag_4_(ThioSila)_2_(THT)_2_] (ThioSila = {Me_2_Si(N-C_6_H_4_-2-SPh)_2_}^2–^, THT = tetra­hydro­thio­phene; Liebing & Merzweiler, 2016 ▸). The silane di­amide complexes [*M*_4_(ThioSila)_2_] comprise a planar Si_2_N_4_ *M*_2_ ring that is structurally closely related to the C_2_N_4_ *M*_2_ ring in the dimeric amidinate complexes.

**Figure 3:**
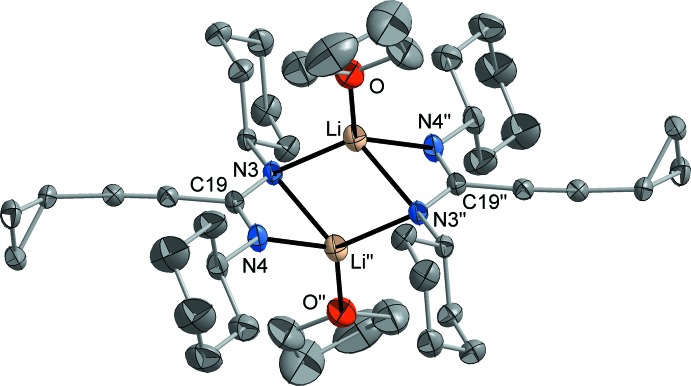
The mol­ecular structure of moiety **2a**, determined from the co-crystal **2**. Displacement ellipsoids are drawn at the 50% probability level and H atoms have been omitted for clarity. [Symmetry code: (′′) 2 − *x*, 1 − *y*, −*z*.]

Lithium moiety **2b** (Fig. 3 ▸): The mol­ecular structure of **2b** was determined from the above-mentioned co-crystal **2** (**2a** × **2b** × toluene). Like the silver components **1** and **2a**, the lithium moiety exists as a centrosymmetric dimer in the crystalline state. However, the mol­ecular structure of **2b** is considerably different, featuring a centrosymmetric Li_2_N_2_ four-membered ring formed by μ-bridging coordination of one of the N atoms (N3). The Li—N distances within this ring are 2.033 (4)–2.261 (4) Å and therefore in the expected range. The coordination number of the mentioned N atom N3 is consequently raised to four and an *sp* ^3^ hybridization fits best to describe the bonding situation. The second N atom of the amidinate ligand (N4) is attached to only one Li atom with a shorter Li—N bond of 2.001 (4) Å, and its coordination environment is trigonal–planar like in the related silver components. Through this μ-κ*N*:κ*N*:κ*N′*-coordination mode of the amidinate ligands, a ‘ladder’ consisting of three four-membered rings is formed. By coordination of a solvent THF mol­ecule, a typical distorted tetra­hedral coordination of the Li atom is completed. Just like in the case of the silver components **1** and **2a**, the C—N bond lengths within the amidinate moiety are very similar with 1.321 (2) and 1.335 (2) Å. The structural motif of ladder-type dimers is typical for this class of compounds and has frequently been observed for most of the previously characterized lithium amidinates and guanidinates (Stalke *et al.*, 1992 ▸; Snaith & Wright, 1995 ▸; Downard & Chivers, 2001 ▸, Brown *et al.*, 2008 ▸).

## Supra­molecular features   

**Figure 4:**
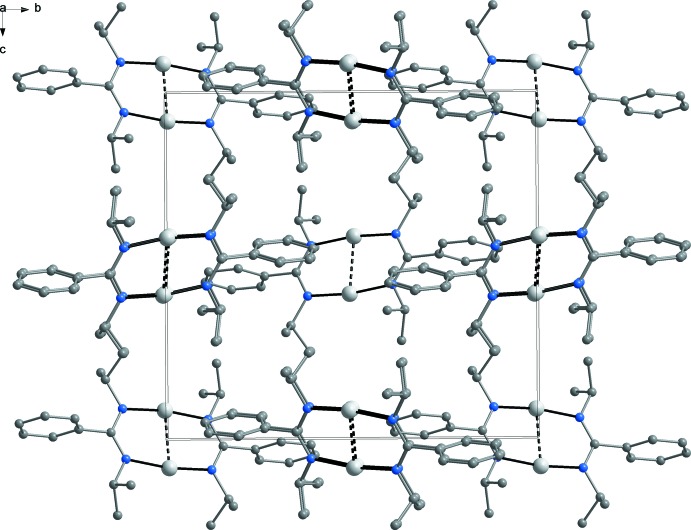
Crystal packing of dimeric silver(I) amidinate mol­ecules in compound **1**, viewed in a projection on (100).

**Figure 5:**
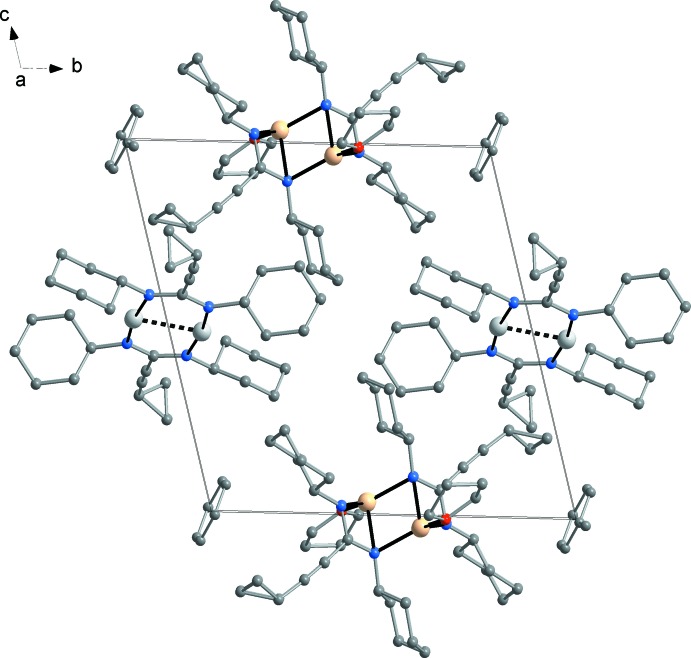
Crystal packing of silver(I) amidinate (**2a**), lithium amidinate (**2b**) and disordered toluene mol­ecules in the co-crystal **2**, viewed in a projection on (100).

In both of the presented crystal structures, there are no specific inter­molecular inter­actions. In compound **1** (Fig. 4 ▸), the closest inter­molecular contacts exist between phenyl groups and isopropyl groups [min. HC⋯CH_3_ 3.79 (1) Å]. In the co-crystal **2** (Fig. 5 ▸), four silver amidinate mol­ecules (**2a**) are situated on the centres of the four unit-cell edges perpendicular to (001) and four lithium amidinate mol­ecules (**2b**) on the four edges perpendicular to (010). The four remaining unit-cell edges perpendicular to (100) are occupied by four disordered toluene mol­ecules. The closest inter­molecular contacts exist between the cyclo­propyl moieties of the silver complex and the toluene methyl groups [C6⋯C44 3.48 (1) Å], followed by cyclo­propyl-cyclo­propyl contacts between silver amidinate and lithium amidinate mol­ecules [C5⋯C24 3.57 (1) Å].

## Synthesis and crystallization   

**Figure 6:**
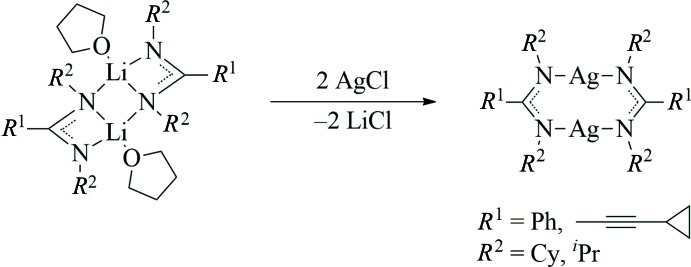
Synthesis of silver(I) amidinates from the related lithium derivatives.

[Ag_2_{PhC(N^*i*^Pr)_2_}_2_] (**1**) was obtained following a published procedure (Lim *et al.*, 2003 ▸). Therefore, an *in situ* prepared solution of the lithium derivative Li{PhC(N^*i*^Pr)_2_} (Sroor *et al.*, 2013 ▸) in THF was treated with a stoichiometric amount of silver(I) chloride at room temperature (Fig. 6 ▸). Afterwards the solvent was removed *in vacuo*, the residue was extracted with toluene and the insoluble matter filtered off. After addition of an excess of *n*-pentane to the filtrate, large colorless crystals formed within few days at room temperature. ^1^H NMR (400.1 MHz, THF-*d_8_*, 298 K): *δ* (p.p.m.) 7.45–7.04 (3×*m*, 10H, C*H* Ph), 3.21 (*sept*, 4H, C*H ^i^*Pr), 1.05 (*d*, 24H, C*H*_3_^*i*^Pr). ^13^C NMR (100.6 MHz, THF-*d_8_*, 298 K): *δ* (p.p.m.) 170.4 (N*C*N), 141.1 (*ipso*-*C* Ph), 128.6 (*C*H Ph), 127.3 (*C*H Ph), 126.7 (*para*-*C*H Ph), 49.3 (*C*H ^*i*^Pr), 28.1 (*C*H_3_^*i*^Pr).

Single crystals of the co-crystal (**2**) with composition [Ag{*c*-C_3_H_5_—C≡C—C(NCy)_2_}]_2_ (**2a**) × [Li{*c*-C_3_H_5_—C≡C—C(NCy)_2_}(THF)]_2_ (**2b**) × toluene were serendipitously obtained in an attempt to prepare the pure silver(I) derivative **2a**. The reaction of the *in situ* prepared lithium compound **2b** (Sroor *et al.*, 2013 ▸) with silver(I) chloride in THF analogous to the procedure described for compound **1** afforded a small qu­antity of colorless co-crystals of (**2**). Mp. = 393 K. ^1^H NMR (400.1 MHz, THF-*d_8_*, 298 K): *δ* (p.p.m.): 3.31–3.40 (*m*, 4H, C*H* Cy), 1.55–1.72 (*m*, 20H, C*H*_2_ Cy), 1.34–1.40 (*m*, 2H, C*H c*-C_3_H_5_), 1.09–1.23 (m, 20H, C*H*_2_ Cy), 0.79–0.83 (*m*, 4H, C*H*_2_ *c*-C_3_H_5_), 0.64–0.68 (*m*, 4H, C*H*_2_ *c*-C_3_H_5_). ^13^C NMR (100.6 MHz, THF-*d_8_*, 298 K): *δ* (p.p.m.) 156.5 (N*C*N), 96.6 (CH—*C*≡C), 69.2 (C≡*C–*-C), 58.8 (*C*H Cy), 38.8 (*C*H_2_, Cy), 26.3 (*C*H_2_ Cy), 8.83 (*C*H_2_ *c*-C_3_H_5_), 0.37 (*C*H *c*-C_3_H_5_).

## Refinement   

Crystal data, data collection and structure refinement details are summarized in Table 1 ▸. All H atoms were fixed geom­etrically and refined using a riding model with *U*_iso_(H) = 1.2*U*_eq_(C). C—H distances in CH_3_ groups were constrained to 0.98 Å, those in CH_2_ groups to 0.99 Å and those in CH groups to 1.00 Å. All CH_3_ groups were refined as freely rotating around the C–C vector.

For compound **2**, the reflection (100) was partly obstructed by the beam stop and was therefore omitted from the refinement. The *U*_ij_ components of the C atoms of the THF mol­ecule (C41–C44) were restrained to be similar for atoms closer than 1.7 Å (SIMU restraint in *SHELXL*; the s.u. applied was 0.01 Å^2^). The toluene mol­ecule (C41–C44) is located on an inversion center. Consequently, the methyl group (C44) and the *para*-H atom (H64) are disordered over two positions and were refined with a constrained site occupancy factor of 0.5. The *ipso*-C and *para*-C atom (C42*A* and C42*B*) were refined to be equal (EXYZ and EADP restraints in *SHELXL*).

## Supplementary Material

Crystal structure: contains datablock(s) compound_1, compound_2. DOI: 10.1107/S2056989016017680/wm5332sup1.cif

Structure factors: contains datablock(s) compound_1. DOI: 10.1107/S2056989016017680/wm5332compound_1sup4.hkl

Structure factors: contains datablock(s) compound_2. DOI: 10.1107/S2056989016017680/wm5332compound_2sup5.hkl

CCDC references: 1515190, 1515191

Additional supporting information:  crystallographic information; 3D view; checkCIF report

## Figures and Tables

**Table 1:** Experimental details

	**1**	**2**
Crystal data		
Chemical formula	[Ag_2_(C_13_H_19_N_2_)_2_]	[Ag_2_(C_18_H_27_N_2_)_2_][Li_2_(C_18_H_27_N_2_)_2_(C_4_H_8_O)_2_]·C_7_H_8_
*M*_r_	622.34	1551.62
Crystal system, space group	Orthorhombic, *P* *b* *c* *a*	Triclinic, *P* 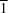
Temperature (K)	153	133
*a*, *b*, *c* (Å)	11.7112 (6), 15.9238 (6), 14.8703 (6)	10.5880 (3), 14.5620 (4), 14.9830 (5)
α, β, γ (°)	90, 90, 90	99.871 (2), 102.825 (2), 106.538 (2)
*V* (Å^3^)	2773.1 (2)	2090.17 (11)
*Z*	4	1
Radiation type	Mo *K*α	Mo *K*α
μ (mm^−1^)	1.43	0.52
Crystal size (mm)	0.23 × 0.21 × 0.09	0.44 × 0.29 × 0.27

Data collection		
Diffractometer	Stoe IPDS 2T	Stoe IPDS 2T
Absorption correction	Numerical (*X-AREA* and *X-RED*; Stoe & Cie, 2002 ▸)	–
*T*_min_, *T*_max_	0.713, 0.874	–
No. of measured, independent and observed [*I* > 2σ(*I*)] reflections	9641, 3026, 2360	22444, 9099, 8214
*R*_int_	0.030	0.043
(sin θ/λ)_max_ (Å^−1^)	0.639	0.639

Refinement		
*R*[*F* ^2^ > 2σ(*F* ^2^)], *wR*(*F* ^2^), *S*	0.024, 0.047, 0.99	0.028, 0.073, 1.03
No. of reflections	3026	9099
No. of parameters	150	461
No. of restraints	0	12
H-atom treatment	H-atom parameters constrained	H-atom parameters constrained
Δρ_max_, Δρ_min_ (e Å^−3^)	0.34, −0.29	0.40, −0.61

Computer programs: *X-AREA* and *X-RED* (Stoe & Cie, 2002 ▸), *SHELXT2013* (Sheldrick, 2015*a* ▸), *SHELXL2016* (Sheldrick, 2015*b* ▸), *DIAMOND* (Brandenburg, 1999 ▸) and *publCIF* (Westrip, 2010 ▸).

## supplementary crystallographic information

### (compound_1) Bis[µ-N1,N2-bis(propan-2-yl)benzamidinato-κ2N1:N2]disilver(I) Crystal data

**Table:**

[Ag_2_(C_13_H_19_N_2_)_2_]	*D*_x_ = 1.491 Mg m^−^^3^
*M**_r_* = 622.34	Mo *K*α radiation, λ = 0.71073 Å
Orthorhombic, *P**b**c**a*	Cell parameters from 11797 reflections
*a* = 11.7112 (6) Å	θ = 2.6–29.1°
*b* = 15.9238 (6) Å	µ = 1.43 mm^−^^1^
*c* = 14.8703 (6) Å	*T* = 153 K
*V* = 2773.1 (2) Å^3^	Plate, colorless
*Z* = 4	0.23 × 0.21 × 0.09 mm
*F*(000) = 1264	

### (compound_1) Bis[µ-N1,N2-bis(propan-2-yl)benzamidinato-κ2N1:N2]disilver(I) Data collection

**Table:**

Stoe IPDS 2T diffractometer	3026 independent reflections
Radiation source: fine-focus sealed tube	2360 reflections with *I* > 2σ(*I*)
Detector resolution: 6.67 pixels mm^-1^	*R*_int_ = 0.030
area detector scans	θ_max_ = 27.0°, θ_min_ = 2.6°
Absorption correction: numerical (*X-AREA* and *X-RED*; Stoe & Cie, 2002)	*h* = −12→14
*T*_min_ = 0.713, *T*_max_ = 0.874	*k* = −17→20
9641 measured reflections	*l* = −18→17

### (compound_1) Bis[µ-N1,N2-bis(propan-2-yl)benzamidinato-κ2N1:N2]disilver(I) Refinement

**Table:**

Refinement on *F*^2^	Secondary atom site location: difference Fourier map
Least-squares matrix: full	Hydrogen site location: inferred from neighbouring sites
*R*[*F*^2^ > 2σ(*F*^2^)] = 0.024	H-atom parameters constrained
*wR*(*F*^2^) = 0.047	*w* = 1/[σ^2^(*F*_o_^2^) + (0.0238*P*)^2^] where *P* = (*F*_o_^2^ + 2*F*_c_^2^)/3
*S* = 0.99	(Δ/σ)_max_ = 0.002
3026 reflections	Δρ_max_ = 0.34 e Å^−^^3^
150 parameters	Δρ_min_ = −0.29 e Å^−^^3^
0 restraints	Extinction correction: SHELXL2016 (Sheldrick, 2015b), Fc^*^=kFc[1+0.001xFc^2^λ^3^/sin(2θ)]^-1/4^
Primary atom site location: heavy-atom method	Extinction coefficient: 0.00133 (9)

### (compound_1) Bis[µ-N1,N2-bis(propan-2-yl)benzamidinato-κ2N1:N2]disilver(I) Special details

**Table:**

Geometry. All esds (except the esd in the dihedral angle between two l.s. planes) are estimated using the full covariance matrix. The cell esds are taken into account individually in the estimation of esds in distances, angles and torsion angles; correlations between esds in cell parameters are only used when they are defined by crystal symmetry. An approximate (isotropic) treatment of cell esds is used for estimating esds involving l.s. planes.

### (compound_1) Bis[µ-N1,N2-bis(propan-2-yl)benzamidinato-κ2N1:N2]disilver(I) Fractional atomic coordinates and isotropic or equivalent isotropic displacement parameters (Å^2^)

**Table:**

	*x*	*y*	*z*	*U*_iso_*/*U*_eq_	
C1	0.89250 (17)	0.84957 (11)	0.52126 (14)	0.0180 (4)	
C2	0.84003 (17)	0.76359 (12)	0.53144 (14)	0.0192 (4)	
C3	0.72930 (18)	0.75332 (14)	0.56240 (15)	0.0269 (5)	
H1	0.684814	0.801041	0.577986	0.032*	
C4	0.6834 (2)	0.67346 (15)	0.57063 (17)	0.0339 (6)	
H2	0.607343	0.666906	0.591646	0.041*	
C5	0.7464 (2)	0.60378 (15)	0.54883 (17)	0.0347 (6)	
H3	0.714312	0.549257	0.554655	0.042*	
C6	0.8568 (2)	0.61370 (14)	0.51837 (17)	0.0347 (6)	
H4	0.901066	0.565694	0.503496	0.042*	
C7	0.9036 (2)	0.69336 (13)	0.50927 (16)	0.0279 (5)	
H5	0.979524	0.699645	0.487771	0.034*	
C8	0.9646 (2)	0.83508 (14)	0.67386 (15)	0.0307 (5)	
H6	0.926464	0.779127	0.668444	0.037*	
C9	1.0893 (3)	0.82211 (19)	0.6933 (2)	0.0519 (8)	
H7	1.097759	0.790334	0.749337	0.062*	
H9	1.126925	0.876767	0.699461	0.062*	
H8	1.124389	0.790784	0.643782	0.062*	
C10	0.9090 (3)	0.8841 (2)	0.7489 (2)	0.0657 (10)	
H12	0.915326	0.852442	0.805200	0.079*	
H10	0.828196	0.893125	0.734664	0.079*	
H11	0.947262	0.938437	0.755509	0.079*	
C11	0.8084 (2)	0.85689 (12)	0.37151 (15)	0.0255 (5)	
H13	0.793586	0.795930	0.382647	0.031*	
C12	0.6952 (2)	0.90348 (17)	0.3702 (2)	0.0431 (7)	
H15	0.648800	0.883280	0.319816	0.052*	
H16	0.709247	0.963797	0.363193	0.052*	
H14	0.654535	0.893414	0.426792	0.052*	
C13	0.8693 (3)	0.86641 (16)	0.28221 (16)	0.0398 (6)	
H19	0.821450	0.843287	0.234130	0.048*	
H18	0.942018	0.836057	0.284187	0.048*	
H17	0.883690	0.926043	0.270543	0.048*	
N1	0.95209 (16)	0.88054 (10)	0.58907 (12)	0.0225 (4)	
N2	0.88089 (15)	0.88965 (10)	0.44393 (12)	0.0194 (4)	
AG	1.04222 (2)	0.99411 (2)	0.58280 (2)	0.01974 (6)	

### (compound_1) Bis[µ-N1,N2-bis(propan-2-yl)benzamidinato-κ2N1:N2]disilver(I) Atomic displacement parameters (Å^2^)

**Table:**

	*U*^11^	*U*^22^	*U*^33^	*U*^12^	*U*^13^	*U*^23^
C1	0.0154 (10)	0.0156 (8)	0.0229 (10)	−0.0005 (7)	0.0023 (8)	−0.0010 (7)
C2	0.0205 (11)	0.0201 (9)	0.0172 (10)	−0.0048 (8)	−0.0028 (8)	0.0025 (8)
C3	0.0226 (13)	0.0312 (10)	0.0269 (11)	−0.0027 (9)	−0.0015 (9)	0.0032 (9)
C4	0.0260 (12)	0.0422 (12)	0.0334 (14)	−0.0165 (10)	−0.0069 (10)	0.0108 (11)
C5	0.0462 (15)	0.0267 (10)	0.0312 (13)	−0.0194 (10)	−0.0126 (12)	0.0066 (9)
C6	0.0454 (15)	0.0207 (10)	0.0381 (14)	−0.0038 (10)	−0.0069 (12)	−0.0004 (10)
C7	0.0317 (13)	0.0213 (9)	0.0308 (13)	−0.0027 (9)	0.0007 (10)	0.0003 (9)
C8	0.0435 (15)	0.0276 (10)	0.0209 (11)	−0.0129 (11)	−0.0057 (11)	0.0074 (9)
C9	0.057 (2)	0.0559 (17)	0.0424 (17)	0.0170 (14)	−0.0130 (15)	0.0194 (14)
C10	0.068 (2)	0.101 (3)	0.0281 (15)	0.0053 (19)	0.0150 (17)	0.0124 (19)
C11	0.0309 (12)	0.0185 (9)	0.0270 (12)	−0.0041 (9)	−0.0118 (10)	−0.0007 (8)
C12	0.0332 (14)	0.0476 (14)	0.0484 (17)	0.0024 (12)	−0.0179 (13)	−0.0024 (13)
C13	0.0567 (18)	0.0383 (12)	0.0243 (12)	−0.0063 (13)	−0.0097 (12)	−0.0104 (10)
N1	0.0273 (9)	0.0213 (7)	0.0190 (9)	−0.0074 (7)	−0.0039 (8)	0.0036 (7)
N2	0.0213 (9)	0.0169 (7)	0.0200 (9)	−0.0028 (7)	−0.0058 (7)	0.0006 (6)
AG	0.02505 (8)	0.01632 (8)	0.01784 (8)	−0.00466 (6)	−0.00495 (6)	0.00110 (6)

### (compound_1) Bis[µ-N1,N2-bis(propan-2-yl)benzamidinato-κ2N1:N2]disilver(I) Geometric parameters (Å, º)

**Table:**

C1—N1	1.322 (3)	C9—H9	0.9800
C1—N2	1.322 (3)	C9—H8	0.9800
C1—C2	1.508 (3)	C10—H12	0.9800
C2—C7	1.383 (3)	C10—H10	0.9800
C2—C3	1.386 (3)	C10—H11	0.9800
C3—C4	1.386 (3)	C11—N2	1.467 (3)
C3—H1	0.9500	C11—C13	1.514 (4)
C4—C5	1.371 (4)	C11—C12	1.520 (3)
C4—H2	0.9500	C11—H13	1.0000
C5—C6	1.379 (4)	C12—H15	0.9800
C5—H3	0.9500	C12—H16	0.9800
C6—C7	1.389 (3)	C12—H14	0.9800
C6—H4	0.9500	C13—H19	0.9800
C7—H5	0.9500	C13—H18	0.9800
C8—N1	1.461 (3)	C13—H17	0.9800
C8—C9	1.502 (4)	N1—AG	2.0959 (16)
C8—C10	1.509 (4)	N2—AG^i^	2.0965 (16)
C8—H6	1.0000	AG—N2^i^	2.0965 (16)
C9—H7	0.9800	AG—AG^i^	2.6604 (3)
			
N1—C1—N2	122.53 (17)	C8—C10—H12	109.5
N1—C1—C2	118.50 (18)	C8—C10—H10	109.5
N2—C1—C2	118.92 (18)	H12—C10—H10	109.5
C7—C2—C3	119.2 (2)	C8—C10—H11	109.5
C7—C2—C1	119.38 (19)	H12—C10—H11	109.5
C3—C2—C1	121.45 (19)	H10—C10—H11	109.5
C2—C3—C4	120.0 (2)	N2—C11—C13	109.64 (19)
C2—C3—H1	120.0	N2—C11—C12	109.90 (19)
C4—C3—H1	120.0	C13—C11—C12	110.5 (2)
C5—C4—C3	120.9 (2)	N2—C11—H13	108.9
C5—C4—H2	119.6	C13—C11—H13	108.9
C3—C4—H2	119.6	C12—C11—H13	108.9
C4—C5—C6	119.3 (2)	C11—C12—H15	109.5
C4—C5—H3	120.4	C11—C12—H16	109.5
C6—C5—H3	120.4	H15—C12—H16	109.5
C5—C6—C7	120.5 (2)	C11—C12—H14	109.5
C5—C6—H4	119.8	H15—C12—H14	109.5
C7—C6—H4	119.8	H16—C12—H14	109.5
C2—C7—C6	120.2 (2)	C11—C13—H19	109.5
C2—C7—H5	119.9	C11—C13—H18	109.5
C6—C7—H5	119.9	H19—C13—H18	109.5
N1—C8—C9	109.4 (2)	C11—C13—H17	109.5
N1—C8—C10	109.8 (2)	H19—C13—H17	109.5
C9—C8—C10	110.4 (2)	H18—C13—H17	109.5
N1—C8—H6	109.1	C1—N1—C8	121.76 (16)
C9—C8—H6	109.1	C1—N1—AG	123.63 (13)
C10—C8—H6	109.1	C8—N1—AG	114.54 (13)
C8—C9—H7	109.5	C1—N2—C11	121.76 (17)
C8—C9—H9	109.5	C1—N2—AG^i^	123.15 (13)
H7—C9—H9	109.5	C11—N2—AG^i^	115.04 (12)
C8—C9—H8	109.5	N1—AG—N2^i^	170.58 (7)
H7—C9—H8	109.5	N1—AG—AG^i^	85.12 (5)
H9—C9—H8	109.5	N2^i^—AG—AG^i^	85.52 (5)
			
N1—C1—C2—C7	−87.4 (3)	N2—C1—N1—AG	−2.7 (3)
N2—C1—C2—C7	90.1 (3)	C2—C1—N1—AG	174.65 (14)
N1—C1—C2—C3	92.7 (3)	C9—C8—N1—C1	122.8 (2)
N2—C1—C2—C3	−89.8 (3)	C10—C8—N1—C1	−116.0 (3)
C7—C2—C3—C4	−0.2 (3)	C9—C8—N1—AG	−54.4 (2)
C1—C2—C3—C4	179.7 (2)	C10—C8—N1—AG	66.9 (3)
C2—C3—C4—C5	0.3 (4)	N1—C1—N2—C11	−176.00 (19)
C3—C4—C5—C6	0.0 (4)	C2—C1—N2—C11	6.6 (3)
C4—C5—C6—C7	−0.4 (4)	N1—C1—N2—AG^i^	1.4 (3)
C3—C2—C7—C6	−0.2 (3)	C2—C1—N2—AG^i^	−175.99 (14)
C1—C2—C7—C6	179.9 (2)	C13—C11—N2—C1	−137.0 (2)
C5—C6—C7—C2	0.5 (4)	C12—C11—N2—C1	101.3 (2)
N2—C1—N1—C8	−179.6 (2)	C13—C11—N2—AG^i^	45.4 (2)
C2—C1—N1—C8	−2.2 (3)	C12—C11—N2—AG^i^	−76.2 (2)

Symmetry code: (i) −*x*+2, −*y*+2, −*z*+1.

### (compound_2) Bis(µ-N1,N2-dicyclohexyl-3-cyclopropylpropynamidinato-κ2N1:N2)disilver(I) bis(µ-N1,N2-dicyclohexyl-3-cyclopropylpropynamidinato-κ3N1,N2:N1)bis(tetrahydrofuran- κO)lithium(I) toluene monosolvate Crystal data

**Table:**

[Ag_2_(C_18_H_27_N_2_)_2_][Li_2_(C_18_H_27_N_2_)_2_(C_4_H_8_O)_2_]·C_7_H_8_	*Z* = 1
*M**_r_* = 1551.62	*F*(000) = 826
Triclinic, *P*1	*D*_x_ = 1.233 Mg m^−^^3^
*a* = 10.5880 (3) Å	Mo *K*α radiation, λ = 0.71073 Å
*b* = 14.5620 (4) Å	Cell parameters from 27393 reflections
*c* = 14.9830 (5) Å	θ = 2.1–29.2°
α = 99.871 (2)°	µ = 0.52 mm^−^^1^
β = 102.825 (2)°	*T* = 133 K
γ = 106.538 (2)°	Rod, colorless
*V* = 2090.17 (11) Å^3^	0.44 × 0.29 × 0.27 mm

### (compound_2) Bis(µ-N1,N2-dicyclohexyl-3-cyclopropylpropynamidinato-κ2N1:N2)disilver(I) bis(µ-N1,N2-dicyclohexyl-3-cyclopropylpropynamidinato-κ3N1,N2:N1)bis(tetrahydrofuran- κO)lithium(I) toluene monosolvate Data collection

**Table:**

Stoe IPDS 2T diffractometer	8214 reflections with *I* > 2σ(*I*)
Radiation source: fine-focus sealed tube	*R*_int_ = 0.043
Detector resolution: 6.67 pixels mm^-1^	θ_max_ = 27.0°, θ_min_ = 2.1°
area detector scans	*h* = −12→13
22444 measured reflections	*k* = −18→18
9099 independent reflections	*l* = −19→19

### (compound_2) Bis(µ-N1,N2-dicyclohexyl-3-cyclopropylpropynamidinato-κ2N1:N2)disilver(I) bis(µ-N1,N2-dicyclohexyl-3-cyclopropylpropynamidinato-κ3N1,N2:N1)bis(tetrahydrofuran- κO)lithium(I) toluene monosolvate Refinement

**Table:**

Refinement on *F*^2^	Primary atom site location: heavy-atom method
Least-squares matrix: full	Secondary atom site location: difference Fourier map
*R*[*F*^2^ > 2σ(*F*^2^)] = 0.028	Hydrogen site location: inferred from neighbouring sites
*wR*(*F*^2^) = 0.073	H-atom parameters constrained
*S* = 1.03	*w* = 1/[σ^2^(*F*_o_^2^) + (0.0398*P*)^2^ + 0.4823*P*] where *P* = (*F*_o_^2^ + 2*F*_c_^2^)/3
9099 reflections	(Δ/σ)_max_ = 0.003
461 parameters	Δρ_max_ = 0.40 e Å^−^^3^
12 restraints	Δρ_min_ = −0.61 e Å^−^^3^

### (compound_2) Bis(µ-N1,N2-dicyclohexyl-3-cyclopropylpropynamidinato-κ2N1:N2)disilver(I) bis(µ-N1,N2-dicyclohexyl-3-cyclopropylpropynamidinato-κ3N1,N2:N1)bis(tetrahydrofuran- κO)lithium(I) toluene monosolvate Special details

**Table:**

Geometry. All esds (except the esd in the dihedral angle between two l.s. planes) are estimated using the full covariance matrix. The cell esds are taken into account individually in the estimation of esds in distances, angles and torsion angles; correlations between esds in cell parameters are only used when they are defined by crystal symmetry. An approximate (isotropic) treatment of cell esds is used for estimating esds involving l.s. planes.

### (compound_2) Bis(µ-N1,N2-dicyclohexyl-3-cyclopropylpropynamidinato-κ2N1:N2)disilver(I) bis(µ-N1,N2-dicyclohexyl-3-cyclopropylpropynamidinato-κ3N1,N2:N1)bis(tetrahydrofuran- κO)lithium(I) toluene monosolvate Fractional atomic coordinates and isotropic or equivalent isotropic displacement parameters (Å^2^)

**Table:**

	*x*	*y*	*z*	*U*_iso_*/*U*_eq_	Occ. (<1)
C1	0.72575 (17)	0.93982 (12)	0.41483 (12)	0.0218 (3)	
C2	0.57831 (19)	0.90959 (12)	0.37327 (13)	0.0246 (3)	
C3	0.45722 (19)	0.89050 (13)	0.34225 (14)	0.0288 (4)	
C4	0.3132 (2)	0.87385 (16)	0.30639 (16)	0.0372 (4)	
H1	0.250264	0.804282	0.293635	0.045*	
C5	0.2590 (3)	0.9540 (2)	0.33985 (19)	0.0530 (6)	
H3	0.166275	0.933659	0.349156	0.064*	
H2	0.325410	1.015476	0.385331	0.064*	
C6	0.2683 (3)	0.9347 (2)	0.24215 (18)	0.0517 (6)	
H4	0.340640	0.984016	0.226610	0.062*	
H5	0.181473	0.902183	0.190427	0.062*	
C7	0.68836 (18)	0.77293 (12)	0.43251 (13)	0.0250 (3)	
H6	0.591076	0.771776	0.420229	0.030*	
C8	0.6996 (2)	0.70587 (14)	0.34709 (15)	0.0364 (5)	
H8	0.796825	0.710104	0.356146	0.044*	
H7	0.670205	0.728808	0.289972	0.044*	
C9	0.6106 (3)	0.59800 (16)	0.3322 (2)	0.0523 (7)	
H9	0.512424	0.592466	0.315540	0.063*	
H10	0.625175	0.555915	0.278802	0.063*	
C10	0.6448 (3)	0.56124 (16)	0.4195 (2)	0.0543 (7)	
H12	0.739052	0.558114	0.431136	0.065*	
H11	0.580377	0.493464	0.409075	0.065*	
C11	0.6355 (2)	0.62794 (16)	0.50555 (18)	0.0436 (5)	
H13	0.665684	0.604587	0.562243	0.052*	
H14	0.538532	0.624002	0.497493	0.052*	
C12	0.7243 (2)	0.73533 (15)	0.52052 (16)	0.0361 (4)	
H16	0.710784	0.777303	0.574501	0.043*	
H15	0.822439	0.740615	0.536326	0.043*	
C13	0.73199 (18)	1.09818 (12)	0.38343 (12)	0.0228 (3)	
H17	0.651330	1.058023	0.327716	0.027*	
C14	0.6799 (2)	1.14824 (14)	0.45820 (14)	0.0304 (4)	
H18	0.758111	1.186566	0.514822	0.037*	
H19	0.614428	1.097199	0.477180	0.037*	
C15	0.6084 (2)	1.21748 (16)	0.42102 (17)	0.0398 (5)	
H21	0.525174	1.178218	0.368054	0.048*	
H20	0.579014	1.251029	0.471881	0.048*	
C16	0.7029 (2)	1.29438 (15)	0.38732 (16)	0.0400 (5)	
H22	0.780227	1.339007	0.441950	0.048*	
H23	0.651693	1.334757	0.359347	0.048*	
C17	0.7593 (2)	1.24632 (14)	0.31411 (15)	0.0350 (4)	
H25	0.826715	1.298382	0.297605	0.042*	
H24	0.683240	1.209012	0.255944	0.042*	
C18	0.8288 (2)	1.17612 (13)	0.35133 (14)	0.0293 (4)	
H27	0.859313	1.143032	0.300877	0.035*	
H26	0.911196	1.214723	0.405123	0.035*	
C19	0.94671 (19)	0.64290 (12)	0.06927 (13)	0.0254 (4)	
C20	0.92323 (19)	0.72135 (13)	0.13212 (13)	0.0261 (4)	
C21	0.90549 (19)	0.78792 (13)	0.17990 (13)	0.0255 (4)	
C22	0.88536 (19)	0.87128 (13)	0.23412 (13)	0.0259 (4)	
H28	0.887959	0.871143	0.301222	0.031*	
C23	0.7900 (2)	0.91816 (15)	0.18509 (15)	0.0342 (4)	
H30	0.744110	0.889465	0.116586	0.041*	
H29	0.734145	0.944081	0.220953	0.041*	
C24	0.9408 (2)	0.97115 (14)	0.21517 (16)	0.0355 (4)	
H31	0.978425	1.029887	0.269721	0.043*	
H32	0.988391	0.975263	0.165338	0.043*	
C25	1.03702 (19)	0.59030 (12)	0.20584 (12)	0.0248 (3)	
H33	1.054854	0.660915	0.237426	0.030*	
C26	0.9230 (2)	0.52439 (15)	0.23685 (15)	0.0338 (4)	
H34	0.840624	0.544594	0.221742	0.041*	
H35	0.897781	0.454947	0.200854	0.041*	
C27	0.9664 (2)	0.53054 (16)	0.34234 (15)	0.0401 (5)	
H36	0.983959	0.598669	0.378675	0.048*	
H37	0.890966	0.485109	0.358858	0.048*	
C28	1.0950 (3)	0.50317 (16)	0.36923 (15)	0.0405 (5)	
H39	1.075296	0.433155	0.337186	0.049*	
H38	1.123447	0.510441	0.438347	0.049*	
C29	1.2107 (2)	0.56912 (18)	0.34099 (15)	0.0417 (5)	
H41	1.291641	0.547196	0.355210	0.050*	
H40	1.237522	0.637984	0.378925	0.050*	
C30	1.1689 (2)	0.56665 (15)	0.23579 (14)	0.0324 (4)	
H42	1.244177	0.615136	0.221918	0.039*	
H43	1.156115	0.500118	0.198041	0.039*	
C31	0.8585 (2)	0.70279 (15)	−0.06282 (14)	0.0359 (4)	
H44	0.882137	0.765597	−0.013568	0.043*	
C32	0.7040 (3)	0.6540 (2)	−0.0967 (2)	0.0536 (6)	
H45	0.669609	0.640626	−0.042284	0.064*	
H46	0.680299	0.589873	−0.142688	0.064*	
C33	0.6331 (4)	0.7193 (3)	−0.1434 (3)	0.0720 (9)	
H48	0.532751	0.683588	−0.167797	0.086*	
H47	0.648538	0.780594	−0.095703	0.086*	
C34	0.6882 (4)	0.7455 (3)	−0.2231 (2)	0.0704 (9)	
H50	0.661290	0.684754	−0.274381	0.084*	
H49	0.646067	0.791608	−0.248707	0.084*	
C35	0.8414 (3)	0.7924 (2)	−0.1931 (2)	0.0610 (7)	
H51	0.867929	0.857776	−0.148375	0.073*	
H52	0.873055	0.803077	−0.249146	0.073*	
C36	0.9122 (3)	0.72721 (18)	−0.14558 (17)	0.0465 (5)	
H54	0.895094	0.665048	−0.192628	0.056*	
H53	1.012784	0.762288	−0.122444	0.056*	
C37	0.6472 (3)	0.4020 (2)	−0.0033 (3)	0.0705 (8)	
H55	0.680375	0.468615	−0.014340	0.085*	
H56	0.613828	0.407387	0.053486	0.085*	
C38	0.5371 (4)	0.3329 (4)	−0.0867 (3)	0.0974 (13)	
H58	0.445435	0.330165	−0.079680	0.117*	
H57	0.546322	0.354115	−0.145022	0.117*	
C39	0.5549 (4)	0.2359 (3)	−0.0907 (3)	0.1037 (14)	
H60	0.505404	0.199991	−0.051531	0.124*	
H59	0.522228	0.194648	−0.156541	0.124*	
C40	0.7025 (4)	0.2623 (2)	−0.0526 (3)	0.0784 (10)	
H62	0.723819	0.214845	−0.016809	0.094*	
H61	0.746114	0.260588	−0.104635	0.094*	
C41	0.5148 (4)	0.9502 (2)	−0.08088 (19)	0.0607 (8)	
H65	0.523425	0.914834	−0.137403	0.073*	
C42A	0.6311 (4)	1.0072 (2)	−0.00946 (18)	0.0573 (7)	0.5
C42B	0.6311 (4)	1.0072 (2)	−0.00946 (18)	0.0573 (7)	0.5
H64	0.719917	1.012313	−0.016020	0.069*	0.5
C43	0.6154 (4)	1.0569 (2)	0.07242 (19)	0.0604 (7)	
H63	0.694287	1.096225	0.123303	0.072*	
C44	0.7893 (6)	1.0222 (4)	−0.0176 (4)	0.0552 (13)	0.5
H67	0.851023	1.031026	0.044882	0.066*	0.5
H68	0.821343	1.080825	−0.041261	0.066*	0.5
H66	0.788893	0.963722	−0.061378	0.066*	0.5
N1	0.77781 (15)	0.87526 (10)	0.44791 (10)	0.0232 (3)	
N2	0.80123 (15)	1.03151 (10)	0.41855 (10)	0.0228 (3)	
N3	0.99709 (17)	0.57779 (11)	0.10369 (10)	0.0262 (3)	
N4	0.9242 (2)	0.63837 (12)	−0.02199 (11)	0.0335 (4)	
AG	0.98689 (2)	0.91010 (2)	0.51539 (2)	0.02283 (5)	
LI	0.9428 (4)	0.4339 (2)	0.0324 (3)	0.0376 (8)	
O	0.75344 (18)	0.35921 (12)	0.00793 (13)	0.0502 (4)	

### (compound_2) Bis(µ-N1,N2-dicyclohexyl-3-cyclopropylpropynamidinato-κ2N1:N2)disilver(I) bis(µ-N1,N2-dicyclohexyl-3-cyclopropylpropynamidinato-κ3N1,N2:N1)bis(tetrahydrofuran- κO)lithium(I) toluene monosolvate Atomic displacement parameters (Å^2^)

**Table:**

	*U*^11^	*U*^22^	*U*^33^	*U*^12^	*U*^13^	*U*^23^
C1	0.0194 (8)	0.0237 (8)	0.0225 (8)	0.0091 (6)	0.0054 (6)	0.0037 (6)
C2	0.0249 (9)	0.0215 (8)	0.0282 (8)	0.0090 (7)	0.0071 (7)	0.0068 (7)
C3	0.0243 (9)	0.0273 (8)	0.0339 (9)	0.0084 (7)	0.0072 (7)	0.0078 (7)
C4	0.0201 (9)	0.0420 (11)	0.0444 (12)	0.0061 (8)	0.0043 (8)	0.0117 (9)
C5	0.0333 (12)	0.0762 (17)	0.0590 (15)	0.0316 (12)	0.0130 (11)	0.0194 (13)
C6	0.0328 (12)	0.0764 (17)	0.0522 (14)	0.0245 (12)	0.0060 (10)	0.0296 (13)
C7	0.0216 (8)	0.0224 (8)	0.0331 (9)	0.0071 (7)	0.0096 (7)	0.0103 (7)
C8	0.0399 (11)	0.0230 (9)	0.0437 (11)	0.0020 (8)	0.0210 (9)	0.0042 (8)
C9	0.0569 (15)	0.0252 (10)	0.0702 (17)	−0.0004 (10)	0.0374 (13)	−0.0006 (10)
C10	0.0546 (15)	0.0255 (10)	0.104 (2)	0.0185 (10)	0.0504 (15)	0.0257 (12)
C11	0.0429 (13)	0.0381 (11)	0.0662 (15)	0.0175 (10)	0.0275 (11)	0.0327 (11)
C12	0.0337 (11)	0.0366 (10)	0.0427 (11)	0.0111 (9)	0.0129 (9)	0.0206 (9)
C13	0.0212 (8)	0.0217 (8)	0.0249 (8)	0.0100 (7)	0.0025 (6)	0.0045 (6)
C14	0.0336 (10)	0.0322 (9)	0.0304 (9)	0.0182 (8)	0.0102 (8)	0.0066 (7)
C15	0.0431 (12)	0.0388 (11)	0.0472 (12)	0.0278 (10)	0.0158 (10)	0.0080 (9)
C16	0.0504 (13)	0.0281 (9)	0.0447 (12)	0.0238 (9)	0.0084 (10)	0.0066 (8)
C17	0.0446 (12)	0.0247 (9)	0.0381 (10)	0.0161 (8)	0.0089 (9)	0.0104 (8)
C18	0.0317 (10)	0.0237 (8)	0.0372 (10)	0.0131 (7)	0.0115 (8)	0.0106 (7)
C19	0.0282 (9)	0.0203 (8)	0.0275 (9)	0.0087 (7)	0.0095 (7)	0.0024 (7)
C20	0.0314 (10)	0.0227 (8)	0.0265 (8)	0.0105 (7)	0.0099 (7)	0.0074 (7)
C21	0.0274 (9)	0.0234 (8)	0.0273 (9)	0.0090 (7)	0.0096 (7)	0.0072 (7)
C22	0.0310 (9)	0.0234 (8)	0.0253 (8)	0.0112 (7)	0.0117 (7)	0.0031 (7)
C23	0.0333 (10)	0.0332 (10)	0.0404 (11)	0.0183 (8)	0.0125 (9)	0.0050 (8)
C24	0.0366 (11)	0.0224 (8)	0.0512 (12)	0.0110 (8)	0.0192 (9)	0.0080 (8)
C25	0.0300 (9)	0.0204 (8)	0.0236 (8)	0.0091 (7)	0.0085 (7)	0.0022 (6)
C26	0.0302 (10)	0.0368 (10)	0.0374 (10)	0.0110 (8)	0.0146 (8)	0.0107 (8)
C27	0.0507 (13)	0.0404 (11)	0.0387 (11)	0.0178 (10)	0.0248 (10)	0.0140 (9)
C28	0.0592 (14)	0.0392 (11)	0.0284 (10)	0.0218 (10)	0.0138 (10)	0.0110 (8)
C29	0.0391 (12)	0.0524 (13)	0.0327 (10)	0.0189 (10)	0.0044 (9)	0.0098 (9)
C30	0.0301 (10)	0.0401 (10)	0.0289 (9)	0.0143 (8)	0.0096 (8)	0.0078 (8)
C31	0.0546 (13)	0.0294 (9)	0.0297 (9)	0.0227 (9)	0.0119 (9)	0.0084 (8)
C32	0.0553 (16)	0.0652 (16)	0.0641 (16)	0.0338 (13)	0.0295 (13)	0.0382 (14)
C33	0.068 (2)	0.096 (2)	0.085 (2)	0.0532 (19)	0.0291 (17)	0.0537 (19)
C34	0.077 (2)	0.088 (2)	0.0618 (17)	0.0413 (19)	0.0155 (16)	0.0424 (17)
C35	0.084 (2)	0.0543 (15)	0.0544 (15)	0.0230 (15)	0.0245 (15)	0.0324 (13)
C36	0.0538 (15)	0.0424 (12)	0.0468 (13)	0.0146 (11)	0.0181 (11)	0.0183 (10)
C37	0.0471 (16)	0.0643 (18)	0.095 (2)	0.0133 (14)	0.0204 (16)	0.0159 (17)
C38	0.0507 (19)	0.154 (4)	0.078 (2)	0.026 (2)	0.0113 (17)	0.028 (2)
C39	0.059 (2)	0.096 (3)	0.104 (3)	−0.016 (2)	0.014 (2)	−0.021 (2)
C40	0.070 (2)	0.0409 (14)	0.098 (2)	−0.0083 (14)	0.0217 (18)	−0.0008 (15)
C41	0.100 (2)	0.0539 (15)	0.0398 (13)	0.0408 (16)	0.0238 (15)	0.0118 (11)
C42A	0.092 (2)	0.0514 (14)	0.0405 (13)	0.0388 (15)	0.0191 (14)	0.0177 (11)
C42B	0.092 (2)	0.0514 (14)	0.0405 (13)	0.0388 (15)	0.0191 (14)	0.0177 (11)
C43	0.090 (2)	0.0562 (15)	0.0389 (13)	0.0335 (15)	0.0151 (14)	0.0113 (11)
C44	0.059 (3)	0.068 (3)	0.045 (3)	0.026 (3)	0.017 (2)	0.021 (2)
N1	0.0202 (7)	0.0207 (7)	0.0289 (7)	0.0077 (6)	0.0051 (6)	0.0073 (6)
N2	0.0196 (7)	0.0214 (7)	0.0281 (7)	0.0098 (6)	0.0036 (6)	0.0070 (6)
N3	0.0341 (8)	0.0220 (7)	0.0235 (7)	0.0120 (6)	0.0087 (6)	0.0031 (6)
N4	0.0515 (11)	0.0293 (8)	0.0265 (8)	0.0233 (8)	0.0118 (7)	0.0069 (6)
AG	0.01875 (7)	0.02032 (7)	0.02912 (8)	0.00771 (5)	0.00391 (5)	0.00737 (5)
LI	0.042 (2)	0.0290 (16)	0.0412 (19)	0.0127 (15)	0.0145 (16)	0.0037 (14)
O	0.0445 (10)	0.0380 (8)	0.0559 (10)	0.0024 (7)	0.0143 (8)	0.0005 (7)

### (compound_2) Bis(µ-N1,N2-dicyclohexyl-3-cyclopropylpropynamidinato-κ2N1:N2)disilver(I) bis(µ-N1,N2-dicyclohexyl-3-cyclopropylpropynamidinato-κ3N1,N2:N1)bis(tetrahydrofuran- κO)lithium(I) toluene monosolvate Geometric parameters (Å, º)

**Table:**

C1—N1	1.329 (2)	C26—H35	0.9900
C1—N2	1.331 (2)	C27—C28	1.515 (3)
C1—C2	1.450 (2)	C27—H36	0.9900
C2—C3	1.194 (3)	C27—H37	0.9900
C3—C4	1.433 (3)	C28—C29	1.517 (3)
C4—C5	1.501 (3)	C28—H39	0.9900
C4—C6	1.505 (3)	C28—H38	0.9900
C4—H1	1.0000	C29—C30	1.531 (3)
C5—C6	1.473 (4)	C29—H41	0.9900
C5—H3	0.9900	C29—H40	0.9900
C5—H2	0.9900	C30—H42	0.9900
C6—H4	0.9900	C30—H43	0.9900
C6—H5	0.9900	C31—N4	1.455 (2)
C7—N1	1.467 (2)	C31—C32	1.512 (4)
C7—C8	1.520 (3)	C31—C36	1.529 (3)
C7—C12	1.526 (3)	C31—H44	1.0000
C7—H6	1.0000	C32—C33	1.536 (3)
C8—C9	1.533 (3)	C32—H45	0.9900
C8—H8	0.9900	C32—H46	0.9900
C8—H7	0.9900	C33—C34	1.501 (4)
C9—C10	1.506 (4)	C33—H48	0.9900
C9—H9	0.9900	C33—H47	0.9900
C9—H10	0.9900	C34—C35	1.497 (5)
C10—C11	1.516 (4)	C34—H50	0.9900
C10—H12	0.9900	C34—H49	0.9900
C10—H11	0.9900	C35—C36	1.540 (4)
C11—C12	1.526 (3)	C35—H51	0.9900
C11—H13	0.9900	C35—H52	0.9900
C11—H14	0.9900	C36—H54	0.9900
C12—H16	0.9900	C36—H53	0.9900
C12—H15	0.9900	C37—O	1.426 (4)
C13—N2	1.472 (2)	C37—C38	1.479 (5)
C13—C18	1.521 (3)	C37—H55	0.9900
C13—C14	1.521 (2)	C37—H56	0.9900
C13—H17	1.0000	C38—C39	1.470 (6)
C14—C15	1.530 (3)	C38—H58	0.9900
C14—H18	0.9900	C38—H57	0.9900
C14—H19	0.9900	C39—C40	1.450 (5)
C15—C16	1.513 (3)	C39—H60	0.9900
C15—H21	0.9900	C39—H59	0.9900
C15—H20	0.9900	C40—O	1.419 (3)
C16—C17	1.522 (3)	C40—H62	0.9900
C16—H22	0.9900	C40—H61	0.9900
C16—H23	0.9900	C41—C42B	1.371 (4)
C17—C18	1.529 (2)	C41—C42A	1.371 (4)
C17—H25	0.9900	C41—C43^ii^	1.388 (5)
C17—H24	0.9900	C41—H65	0.9500
C18—H27	0.9900	C42A—C43	1.383 (4)
C18—H26	0.9900	C42A—C44	1.661 (6)
C19—N4	1.321 (2)	C42B—C43	1.383 (4)
C19—N3	1.335 (2)	C42B—H64	0.9500
C19—C20	1.466 (2)	C43—C41^ii^	1.388 (5)
C19—LI^i^	2.428 (4)	C43—H63	0.9500
C20—C21	1.189 (3)	C44—H67	0.9800
C21—C22	1.437 (2)	C44—H68	0.9800
C22—C23	1.503 (3)	C44—H66	0.9800
C22—C24	1.507 (3)	N1—AG	2.0908 (15)
C22—H28	1.0000	N2—AG^iii^	2.0916 (14)
C23—C24	1.486 (3)	N3—LI	2.033 (4)
C23—H30	0.9900	N3—LI^i^	2.261 (4)
C23—H29	0.9900	N4—LI^i^	2.001 (4)
C24—H31	0.9900	AG—N2^iii^	2.0917 (15)
C24—H32	0.9900	AG—AG^iii^	2.6838 (3)
C25—N3	1.459 (2)	LI—O	1.906 (4)
C25—C30	1.523 (3)	LI—N4^i^	2.001 (4)
C25—C26	1.528 (3)	LI—N3^i^	2.261 (4)
C25—H33	1.0000	LI—C19^i^	2.428 (4)
C26—C27	1.524 (3)	LI—LI^i^	2.440 (7)
C26—H34	0.9900		
			
N1—C1—N2	123.71 (15)	C26—C27—H37	109.4
N1—C1—C2	118.48 (15)	H36—C27—H37	108.0
N2—C1—C2	117.81 (15)	C27—C28—C29	110.48 (17)
C3—C2—C1	175.75 (19)	C27—C28—H39	109.6
C2—C3—C4	176.6 (2)	C29—C28—H39	109.6
C3—C4—C5	118.31 (19)	C27—C28—H38	109.6
C3—C4—C6	118.78 (19)	C29—C28—H38	109.6
C5—C4—C6	58.69 (17)	H39—C28—H38	108.1
C3—C4—H1	116.3	C28—C29—C30	111.95 (18)
C5—C4—H1	116.3	C28—C29—H41	109.2
C6—C4—H1	116.3	C30—C29—H41	109.2
C6—C5—C4	60.81 (16)	C28—C29—H40	109.2
C6—C5—H3	117.7	C30—C29—H40	109.2
C4—C5—H3	117.7	H41—C29—H40	107.9
C6—C5—H2	117.7	C25—C30—C29	112.68 (16)
C4—C5—H2	117.7	C25—C30—H42	109.1
H3—C5—H2	114.8	C29—C30—H42	109.1
C5—C6—C4	60.50 (16)	C25—C30—H43	109.1
C5—C6—H4	117.7	C29—C30—H43	109.1
C4—C6—H4	117.7	H42—C30—H43	107.8
C5—C6—H5	117.7	N4—C31—C32	110.88 (18)
C4—C6—H5	117.7	N4—C31—C36	109.39 (18)
H4—C6—H5	114.8	C32—C31—C36	109.73 (19)
N1—C7—C8	110.92 (14)	N4—C31—H44	108.9
N1—C7—C12	110.30 (15)	C32—C31—H44	108.9
C8—C7—C12	110.42 (16)	C36—C31—H44	108.9
N1—C7—H6	108.4	C31—C32—C33	111.7 (2)
C8—C7—H6	108.4	C31—C32—H45	109.3
C12—C7—H6	108.4	C33—C32—H45	109.3
C7—C8—C9	111.51 (17)	C31—C32—H46	109.3
C7—C8—H8	109.3	C33—C32—H46	109.3
C9—C8—H8	109.3	H45—C32—H46	107.9
C7—C8—H7	109.3	C34—C33—C32	110.7 (2)
C9—C8—H7	109.3	C34—C33—H48	109.5
H8—C8—H7	108.0	C32—C33—H48	109.5
C10—C9—C8	111.6 (2)	C34—C33—H47	109.5
C10—C9—H9	109.3	C32—C33—H47	109.5
C8—C9—H9	109.3	H48—C33—H47	108.1
C10—C9—H10	109.3	C35—C34—C33	112.6 (3)
C8—C9—H10	109.3	C35—C34—H50	109.1
H9—C9—H10	108.0	C33—C34—H50	109.1
C9—C10—C11	111.62 (18)	C35—C34—H49	109.1
C9—C10—H12	109.3	C33—C34—H49	109.1
C11—C10—H12	109.3	H50—C34—H49	107.8
C9—C10—H11	109.3	C34—C35—C36	111.3 (2)
C11—C10—H11	109.3	C34—C35—H51	109.4
H12—C10—H11	108.0	C36—C35—H51	109.4
C10—C11—C12	111.61 (18)	C34—C35—H52	109.4
C10—C11—H13	109.3	C36—C35—H52	109.4
C12—C11—H13	109.3	H51—C35—H52	108.0
C10—C11—H14	109.3	C31—C36—C35	111.1 (2)
C12—C11—H14	109.3	C31—C36—H54	109.4
H13—C11—H14	108.0	C35—C36—H54	109.4
C7—C12—C11	111.45 (18)	C31—C36—H53	109.4
C7—C12—H16	109.3	C35—C36—H53	109.4
C11—C12—H16	109.3	H54—C36—H53	108.0
C7—C12—H15	109.3	O—C37—C38	104.5 (3)
C11—C12—H15	109.3	O—C37—H55	110.8
H16—C12—H15	108.0	C38—C37—H55	110.8
N2—C13—C18	110.41 (14)	O—C37—H56	110.8
N2—C13—C14	111.62 (14)	C38—C37—H56	110.8
C18—C13—C14	109.78 (15)	H55—C37—H56	108.9
N2—C13—H17	108.3	C39—C38—C37	105.6 (3)
C18—C13—H17	108.3	C39—C38—H58	110.6
C14—C13—H17	108.3	C37—C38—H58	110.6
C13—C14—C15	111.17 (16)	C39—C38—H57	110.6
C13—C14—H18	109.4	C37—C38—H57	110.6
C15—C14—H18	109.4	H58—C38—H57	108.8
C13—C14—H19	109.4	C40—C39—C38	102.6 (3)
C15—C14—H19	109.4	C40—C39—H60	111.3
H18—C14—H19	108.0	C38—C39—H60	111.3
C16—C15—C14	111.29 (18)	C40—C39—H59	111.3
C16—C15—H21	109.4	C38—C39—H59	111.3
C14—C15—H21	109.4	H60—C39—H59	109.2
C16—C15—H20	109.4	O—C40—C39	108.0 (3)
C14—C15—H20	109.4	O—C40—H62	110.1
H21—C15—H20	108.0	C39—C40—H62	110.1
C15—C16—C17	111.32 (17)	O—C40—H61	110.1
C15—C16—H22	109.4	C39—C40—H61	110.1
C17—C16—H22	109.4	H62—C40—H61	108.4
C15—C16—H23	109.4	C42B—C41—C43^ii^	121.4 (3)
C17—C16—H23	109.4	C42A—C41—C43^ii^	121.4 (3)
H22—C16—H23	108.0	C42A—C41—H65	119.3
C16—C17—C18	111.25 (17)	C43^ii^—C41—H65	119.3
C16—C17—H25	109.4	C41—C42A—C43	118.0 (3)
C18—C17—H25	109.4	C41—C42A—C44	123.4 (3)
C16—C17—H24	109.4	C43—C42A—C44	118.5 (3)
C18—C17—H24	109.4	C41—C42B—C43	118.0 (3)
H25—C17—H24	108.0	C41—C42B—H64	121.0
C13—C18—C17	111.56 (16)	C43—C42B—H64	121.0
C13—C18—H27	109.3	C42B—C43—C41^ii^	120.6 (3)
C17—C18—H27	109.3	C42A—C43—C41^ii^	120.6 (3)
C13—C18—H26	109.3	C42A—C43—H63	119.7
C17—C18—H26	109.3	C41^ii^—C43—H63	119.7
H27—C18—H26	108.0	C42A—C44—H67	109.5
N4—C19—N3	118.85 (16)	C42A—C44—H68	109.5
N4—C19—C20	120.31 (16)	H67—C44—H68	109.5
N3—C19—C20	120.77 (16)	C42A—C44—H66	109.5
N4—C19—LI^i^	55.46 (13)	H67—C44—H66	109.5
N3—C19—LI^i^	66.70 (13)	H68—C44—H66	109.5
C20—C19—LI^i^	158.53 (15)	C1—N1—C7	119.72 (14)
C21—C20—C19	176.60 (19)	C1—N1—AG	122.90 (11)
C20—C21—C22	177.03 (19)	C7—N1—AG	117.28 (11)
C21—C22—C23	118.85 (16)	C1—N2—C13	119.09 (14)
C21—C22—C24	118.48 (16)	C1—N2—AG^iii^	122.58 (11)
C23—C22—C24	59.15 (13)	C13—N2—AG^iii^	118.05 (11)
C21—C22—H28	116.1	C19—N3—C25	118.58 (14)
C23—C22—H28	116.1	C19—N3—LI	123.43 (16)
C24—C22—H28	116.1	C25—N3—LI	111.73 (15)
C24—C23—C22	60.54 (13)	C19—N3—LI^i^	80.46 (14)
C24—C23—H30	117.7	C25—N3—LI^i^	148.51 (16)
C22—C23—H30	117.7	LI—N3—LI^i^	69.00 (17)
C24—C23—H29	117.7	C19—N4—C31	119.98 (16)
C22—C23—H29	117.7	C19—N4—LI^i^	91.58 (16)
H30—C23—H29	114.8	C31—N4—LI^i^	144.63 (18)
C23—C24—C22	60.31 (13)	N1—AG—N2^iii^	170.66 (5)
C23—C24—H31	117.7	N1—AG—AG^iii^	85.27 (4)
C22—C24—H31	117.7	N2^iii^—AG—AG^iii^	85.45 (4)
C23—C24—H32	117.7	O—LI—N4^i^	118.78 (19)
C22—C24—H32	117.7	O—LI—N3	114.34 (19)
H31—C24—H32	114.9	N4^i^—LI—N3	124.4 (2)
N3—C25—C30	109.55 (15)	O—LI—N3^i^	110.21 (18)
N3—C25—C26	111.24 (15)	N4^i^—LI—N3^i^	64.58 (12)
C30—C25—C26	110.27 (15)	N3—LI—N3^i^	111.00 (17)
N3—C25—H33	108.6	O—LI—C19^i^	112.66 (17)
C30—C25—H33	108.6	N4^i^—LI—C19^i^	32.96 (8)
C26—C25—H33	108.6	N3—LI—C19^i^	129.55 (18)
C27—C26—C25	112.17 (17)	N3^i^—LI—C19^i^	32.85 (7)
C27—C26—H34	109.2	O—LI—LI^i^	131.6 (3)
C25—C26—H34	109.2	N4^i^—LI—LI^i^	94.2 (2)
C27—C26—H35	109.2	N3—LI—LI^i^	59.91 (14)
C25—C26—H35	109.2	N3^i^—LI—LI^i^	51.08 (14)
H34—C26—H35	107.9	C19^i^—LI—LI^i^	75.64 (17)
C28—C27—C26	110.99 (17)	C40—O—C37	109.2 (2)
C28—C27—H36	109.4	C40—O—LI	118.0 (2)
C26—C27—H36	109.4	C37—O—LI	123.21 (19)
C28—C27—H37	109.4		
			
C3—C4—C5—C6	108.2 (2)	C44—C42A—C43—C41^ii^	177.3 (3)
C3—C4—C6—C5	−107.4 (2)	N2—C1—N1—C7	−174.14 (16)
N1—C7—C8—C9	177.97 (19)	C2—C1—N1—C7	6.6 (2)
C12—C7—C8—C9	55.4 (2)	N2—C1—N1—AG	2.2 (2)
C7—C8—C9—C10	−55.3 (3)	C2—C1—N1—AG	−177.07 (12)
C8—C9—C10—C11	54.4 (3)	C8—C7—N1—C1	96.9 (2)
C9—C10—C11—C12	−54.5 (3)	C12—C7—N1—C1	−140.43 (17)
N1—C7—C12—C11	−178.41 (16)	C8—C7—N1—AG	−79.60 (17)
C8—C7—C12—C11	−55.5 (2)	C12—C7—N1—AG	43.06 (18)
C10—C11—C12—C7	55.2 (3)	N1—C1—N2—C13	−177.37 (16)
N2—C13—C14—C15	179.88 (16)	C2—C1—N2—C13	1.9 (2)
C18—C13—C14—C15	57.1 (2)	N1—C1—N2—AG^iii^	−3.6 (2)
C13—C14—C15—C16	−56.7 (2)	C2—C1—N2—AG^iii^	175.62 (12)
C14—C15—C16—C17	54.8 (2)	C18—C13—N2—C1	−153.85 (16)
C15—C16—C17—C18	−54.2 (2)	C14—C13—N2—C1	83.74 (19)
N2—C13—C18—C17	179.80 (15)	C18—C13—N2—AG^iii^	32.10 (18)
C14—C13—C18—C17	−56.7 (2)	C14—C13—N2—AG^iii^	−90.30 (16)
C16—C17—C18—C13	55.6 (2)	N4—C19—N3—C25	172.85 (17)
C21—C22—C23—C24	−107.76 (19)	C20—C19—N3—C25	−4.1 (3)
C21—C22—C24—C23	108.4 (2)	LI^i^—C19—N3—C25	153.26 (19)
N3—C25—C26—C27	175.81 (16)	N4—C19—N3—LI	−37.3 (3)
C30—C25—C26—C27	54.1 (2)	C20—C19—N3—LI	145.79 (19)
C25—C26—C27—C28	−57.2 (2)	LI^i^—C19—N3—LI	−56.9 (2)
C26—C27—C28—C29	56.8 (2)	N4—C19—N3—LI^i^	19.59 (19)
C27—C28—C29—C30	−55.1 (2)	C20—C19—N3—LI^i^	−157.33 (19)
N3—C25—C30—C29	−174.89 (16)	C30—C25—N3—C19	−140.43 (17)
C26—C25—C30—C29	−52.1 (2)	C26—C25—N3—C19	97.41 (19)
C28—C29—C30—C25	53.6 (2)	C30—C25—N3—LI	66.4 (2)
N4—C31—C32—C33	−177.9 (2)	C26—C25—N3—LI	−55.8 (2)
C36—C31—C32—C33	−56.9 (3)	C30—C25—N3—LI^i^	−18.6 (3)
C31—C32—C33—C34	56.0 (4)	C26—C25—N3—LI^i^	−140.7 (2)
C32—C33—C34—C35	−54.5 (4)	N3—C19—N4—C31	174.79 (18)
C33—C34—C35—C36	54.4 (4)	C20—C19—N4—C31	−8.3 (3)
N4—C31—C36—C35	178.0 (2)	LI^i^—C19—N4—C31	−163.3 (2)
C32—C31—C36—C35	56.1 (3)	N3—C19—N4—LI^i^	−21.9 (2)
C34—C35—C36—C31	−55.0 (3)	C20—C19—N4—LI^i^	154.99 (18)
O—C37—C38—C39	26.4 (4)	C32—C31—N4—C19	−90.0 (2)
C37—C38—C39—C40	−32.0 (4)	C36—C31—N4—C19	148.8 (2)
C38—C39—C40—O	26.0 (4)	C32—C31—N4—LI^i^	119.8 (3)
C43^ii^—C41—C42A—C43	0.8 (4)	C36—C31—N4—LI^i^	−1.3 (4)
C43^ii^—C41—C42A—C44	−177.2 (3)	C39—C40—O—C37	−10.2 (4)
C43^ii^—C41—C42B—C43	0.8 (4)	C39—C40—O—LI	−157.6 (3)
C41—C42B—C43—C41^ii^	−0.8 (4)	C38—C37—O—C40	−10.2 (4)
C41—C42A—C43—C41^ii^	−0.8 (4)	C38—C37—O—LI	135.2 (3)

Symmetry codes: (i) −*x*+2, −*y*+1, −*z*; (ii) −*x*+1, −*y*+2, −*z*; (iii) −*x*+2, −*y*+2, −*z*+1.
